# Circulating oxidized low-density lipoproteins and arterial elasticity: comparison between men with metabolic syndrome and physically active counterparts

**DOI:** 10.1186/1475-2840-9-41

**Published:** 2010-08-20

**Authors:** Hanna Pohjantähti-Maaroos, Ari Palomäki, Päivi Kankkunen, Ruth Laitinen, Sari Husgafvel, Kalevi Oksanen

**Affiliations:** 1 Kanta-Häme Central Hospital, Ahvenistontie 20, FI-13530, Hämeenlinna, Finland; 2 Linnan Klinikka, Raatihuoneenkatu 10, FI-13100, Hämeenlinna, Finland; 3 Kuopio University Hospital, PL 1777, FI-70211, Kuopio, Finland

## Abstract

**Background:**

Accumulation of oxidized low-density lipoproteins in the intimae of arteries and endothelial dysfunction are key events in the development of atherosclerosis. Patients with metabolic syndrome are at high risk for cardiovascular diseases but the linkage between metabolic syndrome and atherosclerosis is incompletely understood. We studied whether the levels of oxidized LDL and arterial elasticity differ between metabolic syndrome patients and physically active controls.

**Methods:**

40 men with metabolic syndrome and 40 physically active controls participated in this cross-sectional study. None of the study subjects had been diagnosed with cardiovascular disease. Levels of oxidized LDL were assessed by a two-site ELISA immunoassay. Arterial elasticity was assessed non-invasively by the HDI/PulseWave™ CR-2000 arterial tonometer.

**Results:**

Levels of oxidized LDL were 89.6 ± 33.1 U/L for metabolic syndrome subjects and 68.5 ± 23.6 U/L for controls (p = 0.007). The difference remained significant after adjustment for LDL cholesterol. Large artery elasticity index (C1) was 16.2 ± 4.1 mL/mmHgx10 for metabolic syndrome subjects and 19.4 ± 3.7 mL/mmHgx10 for controls (p = 0.001), small artery indices (C2) were 7.0 ± 3.2 mL/mmHgx100 and 6.5 ± 2.9 mL/mmHgx100 (NS), respectively.

**Conclusions:**

Subjects with metabolic syndrome had elevated levels of oxidized LDL and reduced large arterial elasticity compared to controls. This finding may partly explain the increased risk for cardiovascular diseases among metabolic syndrome patients.

**Trial registration:**

ClinicalTrials.gov NCT01114763

## Background

Metabolic syndrome (MetS) is an accumulation of cardiovascular risk factors: visceral obesity, hypertension, dyslipidemia and abnormal glucose tolerance or diabetes. Subjects with metabolic syndrome are at high risk for cardiovascular diseases [1,2]. Mechanisms that link metabolic syndrome to increased risk are, however, incompletely understood.

The key event in atherogenesis is oxidation of LDL particles entrapped in the intimae of arteries [3]. Elevated levels of oxidized LDL (oxLDL) have been reported to correlate with subclinical atherosclerosis and predict future cardiovascular events [4,5].

Oxidized LDL, together with risk factors known to enhance atherosclerosis, damages the endothelium of the arterial wall [3]. Dysfunction of the endothelium leads into impaired elasticity of the artery already at an early stage of the atherosclerotic process [6]. Aortic stiffness has been found to predict future coronary events and cardiovascular death in previous studies [7,8]. Especially a reduction in the elasticity of small arteries has been found prominent in atherosclerosis and is believed to serve as a marker for early stages of atherosclerosis [6,9].

As a part of Hämeenlinna Metabolic Syndrome Research Program, we studied whether the levels of oxidized LDL and arterial elasticity assessed by a non-invasive radial artery tonometer differ between subjects with metabolic syndrome and their physically active controls. To our knowledge, this is the first time that both the levels of oxLDL and arterial elasticity are reported among the same metabolic syndrome subjects.

## Methods

### Subjects

40 Finnish men with metabolic syndrome diagnosed in routine health examination and laboratory tests, and their 40 age-matched, physically active controls were enrolled in the study. Only men aged 30-65 were included. Subjects with previously diagnosed cardiovascular disease and subjects on cholesterol-lowering medication, ACE-inhibitor or angiotensin-receptor blocker medication were excluded.

Metabolic syndrome was defined according to the National Cholesterol Education Program (NCEP) as the presence of at least three of the following five criteria [10]:

- waist circumference > 102 cm

- serum triglycerides level ≥ 1.7 mmol/L

- serum high density lipoprotein (HDL) cholesterol level < 1.03 mmol/L

- blood pressure ≥ 130/85 mmHg

- plasma glucose level ≥ 6.1 mmol/L or diabetes mellitus

Information on subjects' diseases, medication, smoking habits, alcohol consumption and cardiovascular diseases in family was gathered during a standardized interview. Subjects filled in a questionnaire on their average amount, type and mode of physical exercise per week. We calculated the energy expenditure of mean daily physical exercise in kilocalories by multiplying the MET value and exercise times per week and mean duration of exercise in hours and person's weight in kilograms and finally dividing it by 7 [11]. The compendium of physical activities and subjects' self-rated intensity levels of the exercise sessions were used in estimating the correct MET value [12]. To exclude controls with obstructive cardiovascular disease, participation was accepted if a subject exercised physically more than three times a week and more than 30 minutes per exercise without any symptoms of cardiovascular disease. Mean alcohol intake (g/day) was calculated by multiplying the average number of alcohol portions/month by ethanol content of each taken beverage and dividing it by 30. Subjects' weight, height and waist circumference were measured. Subjects were given both oral and written information on the study before they signed an informed consent. The study was approved by the ethics committee of the Kanta-Häme Hospital District in Finland.

### Laboratory Procedures

Serum levels of total cholesterol, low density lipoprotein (LDL) cholesterol, HDL cholesterol and triglyceride levels were analyzed by a commercial Cobas Integra procedure (Roche). HbA1C was assessed by a standardized method in % and then calculated to mmol/mol according to Nathan et al [13]. Plasma levels of oxidized LDL were determined as duplicates according to a validated, commercial two-site immunoassay (ELISA, Mercodia). The assay has been reported to have an excellent reproducibility [14] and it uses the same monoclonal antibody mAb-4E6 as the assays previously described by Holvoet et al [15].

### Determination of Arterial Elasticity

Arterial elasticity was measured after at least 10 minutes of rest in a semi-sitting position. Subjects refrained from eating, smoking, drinking caffeinated drinks and taking medication for 12 hours and drinking alcohol for two days prior to the measurement. Arterial elasticity was assessed non-invasively by recording radial artery pulse wave by an arterial tonometer (HDI/PulseWave™ CR-2000) which uses a modified Windkessel method [9]. The capacitive elasticity of large arteries (C1) and the reflective elasticity of small arteries (C2) were automatically assessed by the CR-2000 as a mean of five most similar pulse waves appearing during the measurement. C1 identifies the elastic properties of aorta and other large arteries, C2 the endothelial function of the microvascular circulation [6]. Four measurements were performed to gain mean large and small arterial elasticity for every subject. Blood pressure was assessed automatically by the CR-2000 during the elasticity measurement.

### Statistical Methods

Statistics were analyzed with SPSS for Windows 17.0. Differences in continuous variables between metabolic syndrome subjects and controls were studied by Student's T-test in case of normality and by Mann Whitney U-test in case of non-normality. ANOVA was used to analyze the difference in levels of oxidized LDL between the groups after adjustment for LDL and total cholesterol as well as the differences in oxLDL and arterial elasticity after adjustment for the amount of physical activity. Differences in categorical values were calculated by χ^2 ^test. Data are expressed as mean ± SD. A probability value < 0.05 was considered statistically significant.

## Results

The clinical characteristics of the groups are reported in Table 1 and the number of subjects with separate variables of metabolic syndrome defined by NCEP in Table 2. Clinical chemistry is presented in Table 3. Three MetS variables were found in thirteen (32.5%), four variables in twenty-three (57.5%) and five variables in four (10.0%) MetS subjects. Five MetS subjects and one control used beta-blockers, one subject and two controls used aspirin, one subject and one control used calcium channel blockers, and one subject and none of controls used diuretics. Eight subjects and none of controls were on diabetes medication.

**Table 1:** Clinical characteristics

	Metabolic Syndrome (n = 40)	Controls (n = 40)	p value
Age, years	49.8 ± 7.1	50.8 ± 8.1	NS
Family history of, n (%)			
- coronary heart disease	16 (40.0%)	18 (45.0%)	NS
- cerebrovascular disease	8 (20.0%)	6 (15.0%)	NS
Diabetics, n (%)	16 (40.0%)	1 (2.5%)	< 0.001
Diagnosed with hypertension, n (%)	9 (22.5%)	2 (5.0%)	0.048
Smoking			0.001
- current, n (%)	12 (30.0%)	1 (2.5%)	
- former, n (%)	16 (40.0%)	12 (30.0%)	
- never, n (%)	12 (30.0%)	27 (67.5%)	
No of pack-years in smokers	16.1 ± 13.9	8.1 ± 8.3	0.030
Physical activity, kcal/day	233.8 ± 236.4	531.8 ± 274.3	< 0.001
Alcohol intake, g/day	16.9 ± 18.2	8.1 ± 6.1	0.006
BMI, kg/m^2^	31.5 ± 4.7	24.0 ± 1.7	< 0.001
Waist circumference, cm	112.2 ± 12.0	87.8 ± 6.4	< 0.001
Systolic blood pressure, mmHg	139.6 ± 15.8	127.6 ± 9.3	< 0.001
Diastolic blood pressure, mmHg	82.1 ± 9.0	74.3 ± 7.2	< 0.001

Clinical characteristics of metabolic syndrome subjects and controls. Data are presented as mean ± SD if not mentioned otherwise.

**Table 2:** Number of subjects with different MetS variables

	Metabolic Syndrome (n = 40)	Controls (n = 40)
Waist circumference > 102 cm, n (%)	36 (90.0%)	1 (2.5%)
Blood pressure ≥ 130/85, n (%)	40 (100.0%)	12 (30.0%)
HDL cholesterol < 1.03 mmol/L, n (%)	15 (37.5%)	1 (2.5%)
Triglycerides ≥ 1.7 mmol/L, n (%)	36 (90.0%)	2 (5.0%)
Glucose ≥ 6.1 mmol/L or diabetes, n (%)	26 (65.0%)	5 (12.5%)

Number of metabolic syndrome subjects and controls who fulfilled separate variables of metabolic syndrome defined by NCEP [10].

**Table 3:** Laboratory characteristics

	Metabolic Syndrome (n = 40)	Controls (n = 40)	p value
Total cholesterol, mmol/L	5.98 ± 1.0	5.31 ± 0.8	0.001
HDL cholesterol, mmol/L	1.17 ± 0.2	1.68 ± 0.4	< 0.001
LDL cholesterol, mmol/L	3.87 ± 1.0	3.42 ± 0.7	0.020
Triglycerides, mmol/L	2.67 ± 1.5	0.88 ± 0.4	< 0.001
Fasting glucose, mmol/L	6.99 ± 1.8	5.53 ± 0.6	< 0.001
HbA1C,			< 0.001
%	6.18 ± 0.9	5.56 ± 0.2	
mmol/mol	44.1 ± 9.8	37.3 ± 2.2	

Laboratory characteristics of subjects with metabolic syndrome and controls. Data are presented as mean ± SD.

**Figure 1:**
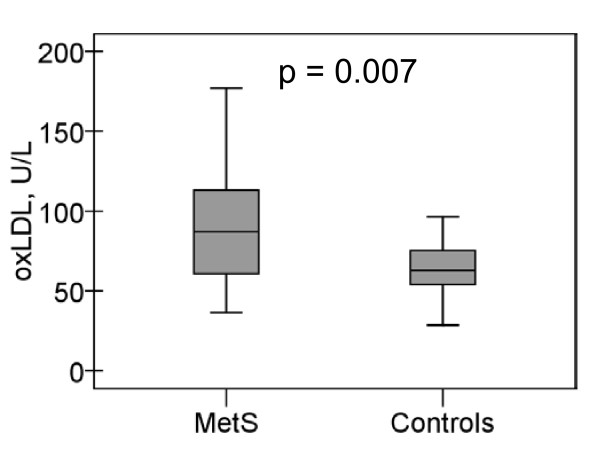
**Oxidized LDL**. Levels of oxidized LDL (U/L) among metabolic syndrome subjects and controls.

Oxidized LDL levels were significantly higher among subjects with MetS than among controls, 89.6 ± 33.1 U/L and 68.5 ± 23.6 U/L (p = 0.007), respectively (Figure 1). The difference remained significant even after adjustment for LDL cholesterol (p = 0.014) and for physical activity (p = 0.015).

**Figure 2:**
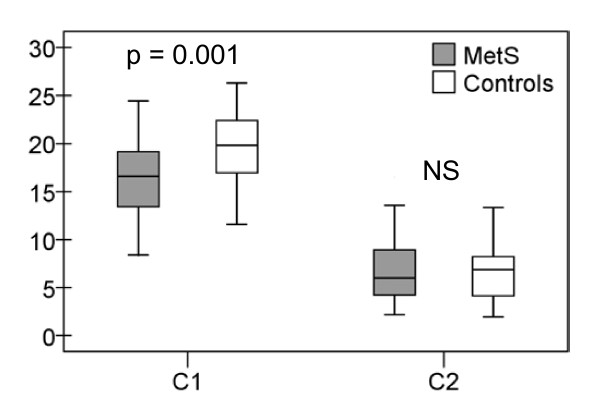
**Arterial elasticity**. Large (C1, mL/mmHgx10) and small (C2, mL/mmHgx100) arterial elasticity among metabolic syndrome subjects and controls.

Large arterial elasticity was significantly better among controls but there was no difference in small arterial elasticity between the groups. Large artery elasticity index (C1) was 16.3 ± 4.1 mL/mmHgx10 for subjects with metabolic syndrome and 19.4 ± 3.7 mL/mmHgx10 for controls (p = 0.001). The difference remained significant after adjustment for physical activity (p = 0.021). Small artery indices (C2) for the metabolic syndrome and control groups were 7.0 ± 3.2 mL/mmHgx100 and 6.7 ± 3.0 mL/mmHgx100, respectively, (NS), (Figure 2).

## Discussion

Oxidized LDL levels were significantly higher and large arterial elasticity lower among men with metabolic syndrome than among physically active controls. Similar findings on increased oxLDL among MetS have previously been published [16-20]. However, in these previous studies MetS definitions or laboratory techniques were different from the ones in the present study. In addition, subjects in the previous studies differed in age, gender or race from the subjects participating in this study.

Our finding disagrees with a previous study by Sjogren et al [21]. They studied 289 subjects of whom 22 had metabolic syndrome. They found no increase in oxLDL levels among MetS subjects compared to a control group of healthy individuals and those with one to two metabolic syndrome factors. A possible reason for different results might be the small number and percentage of MetS subjects in their study.

In our study, subjects with metabolic syndrome were more often smokers, less active physically, and they used more alcohol than controls. Smoking and alcohol consumption have been reported to relate to elevated oxLDL levels, whereas physical activity, for its part, may improve the resistance of LDL against oxidative modification [22-24]. Although these factors may partly contribute to the increased level of oxLDL among MetS subjects, it is noteworthy that the difference in oxLDL remained significant after adjustment for the amount of physical activity.

Elevated levels of oxLDL among MetS subjects may reflect the increased systemic oxidative stress that Hansel et al [25] previously found to associate with metabolic syndrome. Oxidative stress is believed to be substantial in the development of atherosclerosis [26]. The association between elevated levels of oxidized LDL and early development of atherosclerosis has previously been reported [4]. OxLDL also seems to correlate with established coronary heart disease, acute coronary syndrome and atherosclerotic plaque growth [4,5,14,15].

Induced phagocytosis of oxLDL after in vitro stimulation with glucose and insulin has been reported by Sarigianni et al [27]. Since subjects with metabolic syndrome often have elevated levels of glucose and insulin, it may contribute to the progression of atherosclerosis among them. Holvoet et al reported a strong association between circulating oxLDL, metabolic syndrome and increased risk for myocardial infarction [19]. Increased oxLDL among MetS subjects may thus partly explain the connection between metabolic syndrome and increased risk for cardiovascular events. Assessment of oxLDL might be beneficial in risk stratification among patients with metabolic syndrome.

Previous studies implicate that the link between elevated levels of oxidized LDL and metabolic syndrome might be the increased number of small LDL particles more prone to oxidation [20,28]. We did not assess the number of small dense LDL, which was a limitation of this study.

In a study among type 2 diabetics, MetS *per se *was not associated with a reduction in aortic distensibility [29]. We found significantly lower large arterial elasticity among Finnish metabolic syndrome patients than among their physically active controls. In the present study only minority of the subjects had diabetes. In addition, we assessed small and large arterial elasticity by a radial artery pulse wave analysis, which has been thought to provide a more complete understanding of arterial stiffness [30].

The established and widely used measure of regional arterial stiffness, pulse wave velocity, may be inaccurate among subjects with metabolic syndrome, abdominal obesity and diabetes [31].

There are only two previous reports on impaired large arterial elasticity among metabolic syndrome subjects assessed by the same pulse wave analysis as in the present study [32,33]. Because of genetic differences, the results reported among Chinese MetS subjects cannot be generalized to Caucasians [32]. In the other study, impaired large arterial elasticity was found among Caucasian MetS subjects [33]. However, in that study all features of metabolic syndrome were not evaluated since glucose and lipid profiles were not obtained.

Kals et al [34] found reduced elasticity in both small and large arteries and increased oxidative stress among patients with peripheral arterial disease. A study by Morishita et al [35] demonstrated higher oxidized lipoprotein(a) concentrations among patients with coronary heart disease. Lipoprotein(a) concentrations also correlated with pulse wave velocity among hypertensive patients with coronary heart disease and controls. A study by Moreau et al [36] implicated that oxidative stress might be the reason for reduced carotid arterial compliance among sedentary postmenopausal women. Furthermore, Toikka et al [37] showed an association between decreased compliance of ascending aorta and increased levels of a marker for oxidized LDL in otherwise healthy men and in men with borderline hypertension. Our findings are in line with these previous studies since we report for the first time both the elevated levels of oxLDL and decreased large arterial elasticity among same controlled metabolic syndrome subjects.

Subclinical carotid atherosclerosis is associated with metabolic syndrome [38]. Reduction in the elasticity of large arteries, on its part, has been reported to relate to atherosclerosis, future coronary events and cardiovascular mortality [7,39,40]. Reduced large arterial elasticity found among metabolic syndrome subjects may thereby enlighten the connection between metabolic syndrome and the increased cardiovascular risk.

A significant cardiovascular disease may be present among sedentary subjects although not clinically manifested because of lack of physical activity. We wanted to ensure that controls did not have obstructive atherosclerotic disease, like coronary heart disease or peripheral arterial disease. Therefore, participation of controls was accepted if they exercised physically more than three times a week and more than 30 minutes per exercise.

Regular physical exercise is believed to attenuate age-related reduction in large arterial elasticity [41]. In contrast, some previous reports have assessed a greater reduction in central arterial compliance in resistance-trained men compared with sedentary men [42,43]. In these studies, subjects exercised regularly at a vigorous level, as earlier studies have mainly concentrated on subjects who exercised endurance sports at low-intensity. In our study, the control group consisted of men who regularly exercised endurance sports more than three times a week and some even every day. Despite the strenuous level of exercise among most of the controls, their mean large arterial elasticity was much better than among MetS patients. In a previous study, physical activity did not predict arterial stiffness [44]. In the present study, impairment of large arterial elasticity was evident among MetS subjects even after adjustment for physical activity. Therefore, we believe that the amount of exercise as an inclusion criterion of controls would have revealed a manifest cardiovascular disease without interfering with the results.

We found no difference in small arterial elasticity between metabolic syndrome subjects and controls. In line with this finding, metabolic syndrome has previously been reported to selectively impair central pulse wave velocity but not peripheral pulse wave velocity [45]. Small arterial elasticity is often reduced in the presence of hypertension, diabetes and smoking [9,46,47]. In the present study, all these conditions were more often present among metabolic syndrome subjects. Statins, ACE-inhibitor and angiotensin-receptor blocker medications are known to improve endothelial function and thus small arterial elasticity [48-50]. However, subjects with these medications were excluded from the present study. Whether regular physical exercise damages the endothelium in microvasculature and explains the reduction in small arterial elasticity among physically active subjects has not been published.

## Conclusions

Both the elevated levels of oxidized LDL and reduction in large arterial elasticity were found among men with metabolic syndrome when compared to their physically active controls. Our finding may enlighten the connection between increased cardiovascular risk and metabolic syndrome.

## Abbreviations

MetS: metabolic syndrome; oxLDL: oxidized LDL; NCEP: National Cholesterol Education Program; HDL: high density lipoprotein; LDL: low density lipoprotein; C1: large arterial elasticity; C2: small arterial elasticity; BMI: body mass index

## Competing interests

The authors declare that they have no competing interests.

## Authors' contributions

HPM and AP participated in the acquisition of data, design of the study, analysis and drafting of the manuscript. PK, RL and SH made a substantial contribution to acquisition of data and helped in drafting the manuscript, KO participated in the design of the study and gave final approval of the version to be published. All authors have read and approved the final manuscript.
